# Association between Doppler assessment and secondary cesarean delivery for intrapartum fetal compromise in small-for-gestational-age fetuses

**DOI:** 10.1007/s00404-024-07559-2

**Published:** 2024-05-24

**Authors:** Anna S. Scholz, Vanessa Rónay, Markus Wallwiener, Herbert Fluhr, Alexandra von Au, Julia Spratte, Stephanie Wallwiener, Michael Elsaesser

**Affiliations:** 1 Department of Gynecology and Obstetrics, Heidelberg University Hospital, Im Neuenheimer Feld 440, 69120 Heidelberg, Germany; 2 Department of Urology, Nuernberg Hospital, Nuernberg, Germany; 3 Department of Gynecology, University Hospital Halle (Saale), Halle, Germany; 4 Department of Gynecology and Obstetrics, Graz University Hospital, Graz, Austria; 5 Department of Obstetrics and Fetal Medicine, University Hospital Halle (Saale), Halle, Germany

**Keywords:** Small-for-gestational age, Intrapartum fetal compromise, Cesarean section, Umbilical vein flow, Cerebroplacental ratio, Fetal aorta, Myocardial performance index

## Abstract

**Purpose:**

To elucidate the association between arterial and venous Doppler ultrasound parameters and the risk of secondary cesarean delivery for intrapartum fetal compromise (IFC) and neonatal acidosis in small-for-gestational-age (SGA) fetuses.

**Methods:**

This single-center, prospective, blinded, cohort study included singleton pregnancies with an estimated fetal weight (EFW) < 10th centile above 36 gestational weeks. Upon study inclusion, all women underwent Doppler ultrasound, including umbilical artery (UA) pulsatility index (PI), middle cerebral artery (MCA) PI, fetal aortic isthmus (AoI) PI, umbilical vein blood flow (UVBF), and modified myocardial performance index (mod-MPI). Primary outcome was defined as secondary cesarean section due to IFC.

**Results:**

In total, 87 SGA pregnancies were included, 16% of which required a cesarean section for IFC. Those fetuses revealed lower UVBF corrected for abdominal circumference (AC) (5.2 (4.5–6.3) vs 7.2 (5.5–8.3), p = 0.001). There was no difference when comparing AoI PI, UA PI, ACM PI, or mod-MPI. No association was found for neonatal acidosis. After multivariate logistic regression, UVBF/AC remained independently associated with cesarean section due to IFC (aOR 0.61 [0.37; 0.91], p = 0.03) and yielded an area under the curve (AUC) of 0.78 (95% CI, 0.67–0.89). A cut-off value set at the 50th centile of UVBF/AC reached a sensitivity of 86% and specificity of 58% for the occurrence of cesarean section due to IFC (OR 8.1; 95% CI, 1.7–37.8, p = 0.003).

**Conclusion:**

Low levels of umbilical vein blood flow (UVBF/AC) were associated with an increased risk among SGA fetuses to be delivered by cesarean section for IFC.

**Supplementary Information:**

The online version contains supplementary material available at 10.1007/s00404-024-07559-2.

## Introduction

Small-for-gestational age (SGA) characterizes fetal growth below a certain threshold and these fetuses are often considered as constitutionally small, but healthy [1]. However, even SGA fetuses in which fetoplacental Doppler findings are normal are at increased risk for stillbirth and perinatal mortality and are associated with a higher incidence of cesarean section for intrapartum fetal distress [1, 2]. This evidence highlights that there might be a subgroup of SGA fetuses that does suffer from impaired fetal growth and remains undetected by standard biophysical measurements. Umbilical artery (UA) Doppler as a standalone marker frequently fails to identify placental insufficiency in SGA fetuses near term [3], emphasizing the need for a more detailed risk stratification among SGA fetuses.

Several Doppler assessments of new vascular systems, such as the umbilical vein [4–7], fetal aortic isthmus [8], and cardiac function [9–11], have been proposed to be associated with adverse perinatal outcomes in late-onset growth-restricted fetuses. Reduced umbilical vein blood flow (UVBF) was shown to precede relevant changes in fetal size or UA Doppler waveforms [12]. Additionally, histological analyses by Parra-Saavedra et al. suggested that UVBF is a surrogate for placental underperfusion and damage in SGA fetuses near term [13].

Systematic prenatal assessment of the susceptibility of a fetus to gradually develop hypoxia, which may necessitate undertaking measures to deliver the baby operatively, is yet to be established but would be crucial in order to provide individualized maternity care and advice on the mode of delivery. Unlike previously published studies, we aimed to explore and integrate both standard and extended biophysical Doppler assessments in one cohort.

Therefore, we conducted a blinded, prospective, observational cohort study to evaluate and compare Doppler parameters of the fetal peripheral and central circulation, and to correlate these with the risk of cesarean section due to intrapartum fetal compromise (IFC) in SGA fetuses. Secondly, the Doppler parameters were examined according to neonatal acidosis.

## Methods

### Study design and population

We performed a single-center, blinded, prospective, observational cohort study at the Department of Gynecology and Obstetrics, University Hospital Heidelberg, Germany after ethical approval was obtained by the local Ethics Committee of the Ruprecht-Karls University Heidelberg (S-627/2014). We screened pregnant women above 36 weeks of gestation with an estimated fetal weight (EFW) < 10th centile. We excluded twin pregnancies and those showing fetal cardiac or chromosomal anomalies. All participants provided written informed consent. Upon study inclusion and before onset of labor, all women received an ultrasound assessment. Both women and clinicians were blinded to the ultrasound measurements. Further management of labor and delivery followed local protocols and national guidelines. The study adheres to the STROBE guideline for observational cohort studies, and all methods were performed in accordance with the Declaration of Helsinki.

### Doppler assessment

Ultrasound examinations were performed once before onset of labor by one experienced prenatal sonographer (M.E., DEGUM II level) with a Voluson machine (GE Healthcare, Buckinghamshire, UK). Median gestational age at examination was 38 + 1 weeks. Median time interval between examination and delivery was 2 days. Fetal weight was estimated using the Hadlock-4 formula [14]. Amniotic fluid volume was assessed by calculating the deepest vertical pocket. The following vessels were examined: umbilical vein (UV) and artery (UA), fetal aortic isthmus, middle cerebral artery (MCA), and the modified myocardial performance index (mod-MPI). The cerebroplacental ratio (CPR) was calculated by dividing MCA PI by UA PI. The reference values were provided by ViewPoint (ViewPoint, GE Healthcare, Weβling, Germany).

The umbilical vein blood flow (UVBF) was determined during a period of fetal inactivity from a free-floating part of the umbilical cord [15]. After appropriate image magnification, the inner diameter of the UV was measured. For measuring UVBF velocity (cm/sec) only the smallest possible angle of insonation (< 30°) was accepted and, when an angle of 0° could not be achieved, the angle correction function was used. The UVBF was calculated according to the formula UVBF (ml/min) = UV area (cm^2^) x UVBF velocity (cm/sec) × 0.5 × 60 and was corrected for estimated fetal weight UVBF/EFW [ml/min/kg]) and for abdominal circumference (UVBF/AC) [16]. The venous-arterial index (VAI) was computed by dividing UVBF/EFW by UA PI as described by Tchirikov et al. [17].

The mod-MPI was calculated to evaluate fetal left cardiac function [18, 19]. The Doppler gate was placed on the lateral wall of the ascending aorta, above the mitral valve (MV) and below the aortic valve (AV) towards the ventricular septum. Based on the pulsed Doppler recording, three time periods were calculated: isovolumetric contraction time (ICT) between the closure of the MV and opening of the AV, isovolumetric relaxation time (IRT) between the closure of the AV and opening of the MV, and ejection time (ET) from opening to closure of the AV. The mod-MPI was finally calculated as mod-MPI = (ICT + IRT)/ET.

### Outcome measures

Study participants were followed up until delivery. Patient records were screened for mode of delivery and neonatal outcome. The primary outcome was a secondary cesarean section due to IFC. During labor, fetal heart rate was monitored continuously and the tracings were evaluated by the attending obstetric caregivers. The classification of IFC was made retrospectively by the study team by either pathological fetal hart rate patterns and/or abnormal fetal scalp analysis (pH < 7.2). The secondary outcome measure was neonatal acidosis, defined as arterial umbilical pH ≤ 7.20.

### Statistical analysis

Statistical analysis was performed using Prism 9.5.0 (GraphPad Prism Software, Inc., Diego, CA, USA) and MedCalc 20.218 (MedCalc Software, Ostende, Belgium). Patients were categorized in two groups according to the primary and secondary outcome measure. Continuous data are presented as median (interquartile ranges (IQR)) and categorical data as absolute and relative numbers and were compared using nonparametric Mann-Whitney U and Fisher Yates test, respectively. Two-sided *p* values < 0.05 were considered significant. To account for multiple comparisons, statistical analyses of Doppler parameters were adjusted according to Bonferroni (α < 0.05/9). Univariate logistic regression models were calculated for baseline characteristics and those significant Doppler parameters identified in the multiple comparisons. Multivariable logistic regression modelling includes factors that revealed a p value below 0.1 in univariate analysis. To test for collinearity, variance inflation factor was used. Receiver-operating curve (ROC) analyses including the area under the curve (AUC) were calculated to evaluate the predictive value. Logistic regression analysis was used to evaluate the additive value of combined Doppler parameters, and their ROC curves were compared as described by DeLong et al. [20]. Percentiles of UVBF/AC were set based on the study cohort. Odds ratios (OR) and 95% confidence intervals [CI] were calculated using the Baptista-Pike method. Relationship between variables was evaluated by computing correlation coefficients (Spearman).

## Results

**Fig. 1:**
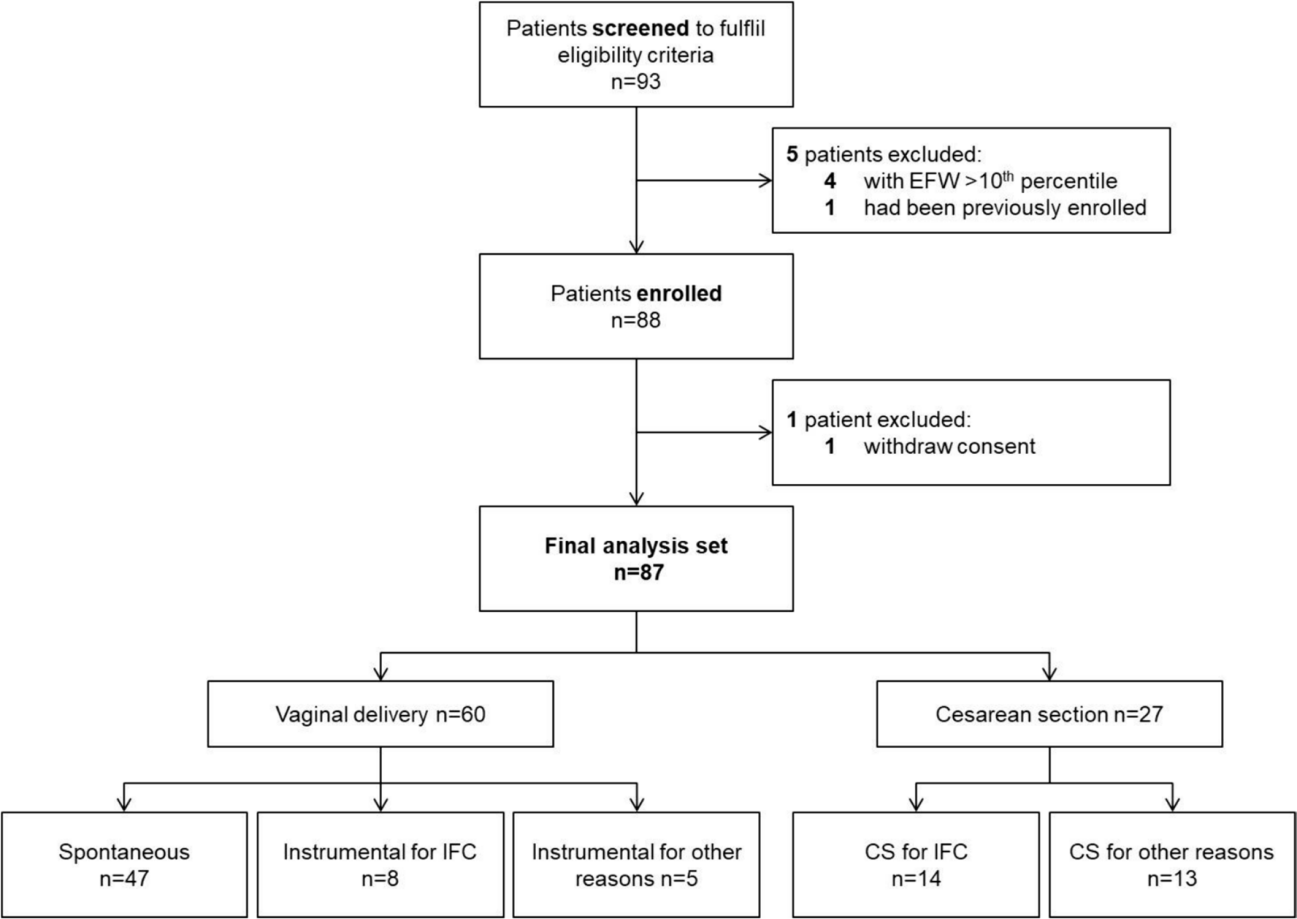
Flow diagram of the study cohort. *IFC* intrapartum fetal compromise, *CS* Cesarean section

A total of 93 patients were screened for eligibility. Of those, four patients were not eligible due to an EFW > 10th centile, one patient had previously been enrolled during her previous pregnancy, and one patient withdrew consent, leaving 87 patients for the final analysis data set (Fig. 1).

Cesarean section for IFC was performed in 14 of 87 deliveries (16%). Baseline characteristics of the study cohort are reported in Table 1. For fetuses of women who required cesarean section for IFC, both AC and EFW as well as birth weight were more likely to be lower.

**Table 1:** Clinical baseline characteristics of the cohort

Variable	Analysis set	Cesarean section for IFC		p value
		No	Yes	
	n = 87	n = 73	n = 14	
Maternal age (years)	29 (25–34)	29 (26–34)	29 (21–36)	0.799
BMI (kg/m^2^)	21 (20–24)	21 (19–23)	23 (21–25)	0.052
Gestational age at examination (days)	267 (265–270)	267 (265–270)	267 (265–271)	0.834
Gestational age at delivery (days)	269 (267–273)	269 (267–273)	271 (267–274)	0.807
Caucasian	73 (84)	60 (82)	13 (93)	
Medical history, n (%)				
Nulliparous	58 (67)	46 (63)	12 (86)	0.128
Active smoking	14 (16)	13 (18)	1 (7)	0.451
Gestational diabetes	8 (9)	7 (10)	1 (7)	> 0.999
HDP	6 (7)	5 (7)	1 (7)	> 0.999
Pregnancy cholestasis	3 (3)	3 (4)	0	> 0.999
Medication, n (%)				
Methyldopa	4 (5)	4 (5)	0	> 0.999
Betablocker	1 (1)	1 (1)	0	> 0.999
Aspirin	3 (3)	3 (4)	0	> 0.999
Baseline sonografic parameters				
Abdominal circumference	300.2 (290–306.2)	301.6 (291.6–308.1)	293.1 (282.7–302.8)	**0.031**
Estimated fetal weight (g)	2474 (2287–2591)	2522 (2324–2608)	2326 (2191–2467)	**0.003**
Single deepest pocket (cm)	3.1 (2.7–3.8)	3 (2.6–3.7)	3.2 (2.9; 3.8)	0.458
Birth weight (g)	2480 (2320–2700)	2530 (2345–2765)	2345 (2038–2523)	**0.010**

Data are presented as median (interquartile range) or as absolute numbers (percentages). p values refer to comparison between those with and without cesarean section due to intrapartum fetal compromise (IFC). Boldface indicates p values < 0.05. Continuous data were compared using Mann-Whitney U test. Categorical variables were compared using Fisher exact test

*BMI* Body mass index, *HDP* hypertensive disorders of pregnancy, *PI* pulsatility index

When stratifying patients for the occurrence of cesarean delivery for IFC, we found significantly lower absolute UVBF (153.6 (134–180.7) vs 220.3 ml/min (169.3–244.4), p = 0.0002) and lower venous-arterial index (VAI) (60.8 (49.6–87.8) vs 94.7 (70–120.4), p = 0.002) in fetuses requiring cesarean delivery for IFC compared to any other delivery (Table 2). The UVBF remained significantly lower in these fetuses even after correcting for both EFW (65.4 (55.8–78) vs 85.4 ml/min/kg (68.4–103.4), p = 0.005) and AC (5.2 (4.5–6.3) vs 7.2 (5.5–8.3), p = 0.001). After exclusion of fetuses delivered by instrumental vaginal delivery for IFC (column “vs. any delivery without IFC”) or any operative delivery (column “vs. spontaneous vaginal delivery”), absolute UVBF, UVBF/AC, and VAI remained significantly lower in fetuses that required cesarean section for IFC. In contrast, no significant differences were observed in CPR, fetal aortic isthmus PI (AoI PI), mod-MPI, UA PI, or MCA PI (Table 2).

**Table 2:** Fetal Doppler parameters according to the mode of delivery and intrapartum fetal compromise

Outcome	CS for IFC	vs. any other delivery		vs. any delivery without IFC		vs. CS for other reasons		vs. spontaneous vaginal delivery	
	n = 14	n = 73	p value	n = 65	p value	n = 13	p value	n = 52	p value
UVBF absolute	153.6	220.3	**0.0002**	220.3	**0.0003**	222.3	0.007	217.8	**0.0004**
	(134–180.7)	(169.3–244.4)		(169.3–241)		(172.4–263.8)		(163–241)	
UVBF/EFW	65.4	85.4	**0.005**	86.5	0.007	86.5	0.054	86.1	0.007
	(55.8–78)	(68.4–103.4)		(67.21–103.4)		(67.1–108)		(67.2–103.7)	
UVBF/AC	5.2	7.2	**0.001**	7.2	**0.0008**	7.51	0.008	7.1	**0.001**
	(4.5–6.3)	(5.5–8.3)		(5.5–8.3)		(5.62–8.97)		(5.5–8.4)	
VAI	60.8	94.7	**0.002**	93.5	**0.002**	93.5	0.061	92.8	**0.002**
	(49.6–87.8)	(70–120.4)		(68–115.4)		(60.9–114.2)		(70.8–122.8)	
AoI PI	1.84	1.78	0.373	1.78	0.41	1.71	0.2	1.79	0.553
	(1.7–1.06)	(1.57–2.01)		(1.56–2.02)		(1.51–1.92)		(0.57–2.05)	
UA PI	1.05	0.9	0.044	0.92	0.067	0.92	0.53	0.9	0.042
	(0.87–1.32)	(0.79–1.1)		(0.79–1.06)		(0.88–1.06)		(0.77–1.06)	
MCA PI	1.15	1.37	0.084	1.39	0.093	1.29	0.224	1.4	0.099
	(1.03–1.54)	(1.15–1.62)		(1.15–1.62)		(1.12–1.59)		(1.15–1.63)	
CPR	1.15	1.57	0.0068	1.5	0.011	1.45	0.144	1.54	0.009
	(0.92–1.45)	(1.21–1.86)		(1.21–1.86)		(1.19–1.75)		(1.22–1.86)	
mod-MPI	0.65	0.6	0.071	0.6	0.066	0.7	0.94	0.59	0.021
	(0.60–0.79)	(0.55–0.71)		(0.55–0.71)		(0.58–0.79)		(0.54–0.67)	

Data are presented as median (interquartile range). p values were calculated using Fisher exact test compared to CS for IFC. To adjust for multiple comparisons, Bonferroni correction was applied for each column. Alpha values < 0.0056 (p < 0.05/9) were considered statistically significant (indicated by boldface)

*AC* abdominal circumference; AoI: aortic isthmus, *CPR* cerebroplacental ratio, *CS* cesarean section, *EFW* estimated fetal weight, *IFC* intrapartum fetal compromise, *MCA* middle cerebral artery, *mod-MPI* modified myocardial performance index, *PI* pulsatility index, *UA* umbilical artery, *UVBF* umbilical vein blood flow, *VAI* venous-arterial index (UVBF [ml/min/kg]/UA PI)

Subgroup analysis by mode of delivery did not reveal a statistically significant association of any Doppler parameters with instrumental vaginal delivery for IFC (Supplemental Table 1). Both absolute UVBF and UVBF/AC tended to be lower in fetuses delivered by any operative delivery for IFC compared to any mode of delivery without IFC.

Overall, 28% of the neonates presented with acidosis (arterial pH ≤ 7.2), and 8% exhibited an APGAR after 5 min ≤ 7. Of those, five neonates suffered severe acidosis, with an umbilical artery pH ≤ 7.1. There was no difference in fetal Doppler parameters according to neonatal acidosis (Table 3).

**Table 3:** Fetal Doppler parameters according to neonatal acidosis

	Umbilical artery pH ≤ 7.2		
Outcome	Yes (n = 24)	No (n = 63)	p value
UVBF absolute	199.6 (154.5–246.8)	204.5 (156.5–239.0)	0.775
UVBF/EFW	77.29 (66.99–100.9)	84.18 (64.73–98.15)	0.935
UVBF/AC	6.71 (5.39–8.39)	6.91 (5.19–7.8)	0.68
VAI	87.96 (61.39–139.1)	90.88 (61.38–113.2)	0.847
AoI PI	1.78 (1.62–2)	1.78 (1.57–2.01)	0.983
UA PI	0.92 (0.75–1.11)	0.92 (08–1.06)	0.632
MCA PI	1.33 (1.05–1.66)	1.32 (1.09–1.58)	0.860
CPR	1.55 (1.07–1.86)	1.45 (1.19–1.76)	0.754
mod-MPI	0.60 (0.58–0.7)	0.62 (0.56–0.72)	0.968

Data are presented as median (interquartile range). p values were calculated using Fisher exact test

*AC* abdominal circumference, *AoI* aortic isthmus, *CPR* cerebroplacental ratio, *EFW* estimated fetal weight, *MCA* middle cerebral artery, *mod-MPI* modified myocardial performance index, *PI* pulsatility index, *UVBF* umbilical vein blood flow, *VAI* venous-arterial index (UVBF [ml/min/kg]/UA PI)

Univariately associated maternal and neonatal characteristics with cesarean section due to IFC are listed in Table 4. In multivariate logistic regression analysis, UVBF/AC was entered as continuous variable and remained independently associated with the risk of cesarean delivery for IFC after adjustment for EFW and maternal weight before pregnancy (aOR 0.61 [0.37; 0.91], p = 0.03). In a separate multivariate model, VAI was also inversely associated with the risk of CS for IFC (aOR 0.97 [0.95; 0.99], p = 0.03) (Table 4). We next aimed to elucidate the discriminative power of the different Doppler parameters to predict cesarean section for IFC. ROC analyses revealed significant discriminations for UVBF/AC with an AUC of 0.78 (95% CI, 0.67- 0.89), demonstrating greater accuracy than that of CPR (0.73; 95% CI, 0.59- 0.87) and VAI (AUC 0.76 (95% CI 0.64–0.88), p = 0.002). Adding CPR to either UVBF/AC or VAI did not significantly increase the AUC compared to CPR alone (∆AUC_UVBF/AC_ = 0.05, p = 0.38; ∆AUC_VAI_ = 0.04, p = 0.29).

**Table 4:** Univariate and multivariate logistic regression analysis for prediction of cesarean section due to IFC

Variable	OR [95% CI]	p value	aOR*[95% CI]	p value	aOR^†^[95% CI]	p value
UVBF absolute	0.98 [0.96; 0.99]	0.003				
UVBF/EFW	0.96 [0.92; 0.99]	0.011				
UVBF/AC	0.54 [0.33; 0.79]	0.005	0.61 [0.37; 0.91]	0.03		
VAI	0.97 [0.95; 0.99]	0.006			0.97 [0.95; 0.99]	0.03
Maternal age (years)	0.98 [0.89; 1.08]	0.69				
Maternal weight before pregancy (kg)	1.05 [0.996; 1.11]	0.07	1.05 [0.99; 1.11]	0.14	1.04 [0.98; 1.11]	0.15
Nulliparous	3.52 [0.87; 23.73]	0.12				
Gestational age at delivery (days)	0.99 [0.89; 1.1]	0.84				
Smoker	0.36 [0.02; 2.0]	0.34				
Gestational diabetes	0.73 [0.04; 4.59]	0.77				
HDP	1.05 [0.05; 7.23]	0.97				
EFW (g)	0.996 [0.99; 0.99]	0.01	0.997 [0.99; 1]	0.05	0.996 [0.99; 1]	0.04
AC	0.95 [0.90; 0.99]	0.03				
Birth weight (g)	0.997 [0.995; 0.99]	0.02				

Data are expressed as odds ratios (OR) with 95%CI. Multivariable logistic regression modeling included factors that revealed a p value below 0.1 in univariate analysis. To avoid collinearity we chose UVBF/AC over absolute UVBF and UVBF/EFW, as UVBF/AC reached statistical significance in all comparisons as seen in Table 2. To avoid further redundancy only one criterion for fetal biometry was included. We chose EFW over birth weight and AC, as we aimed to determine prenatal risk assessment and because AC was already included in UVBF/AC. We calculated two separate multivariate models, one ^*^ included UVBF/AC, the other ^**†**^ included VAI instead because the two parameters showed high collinearity

**Fig. 2:**
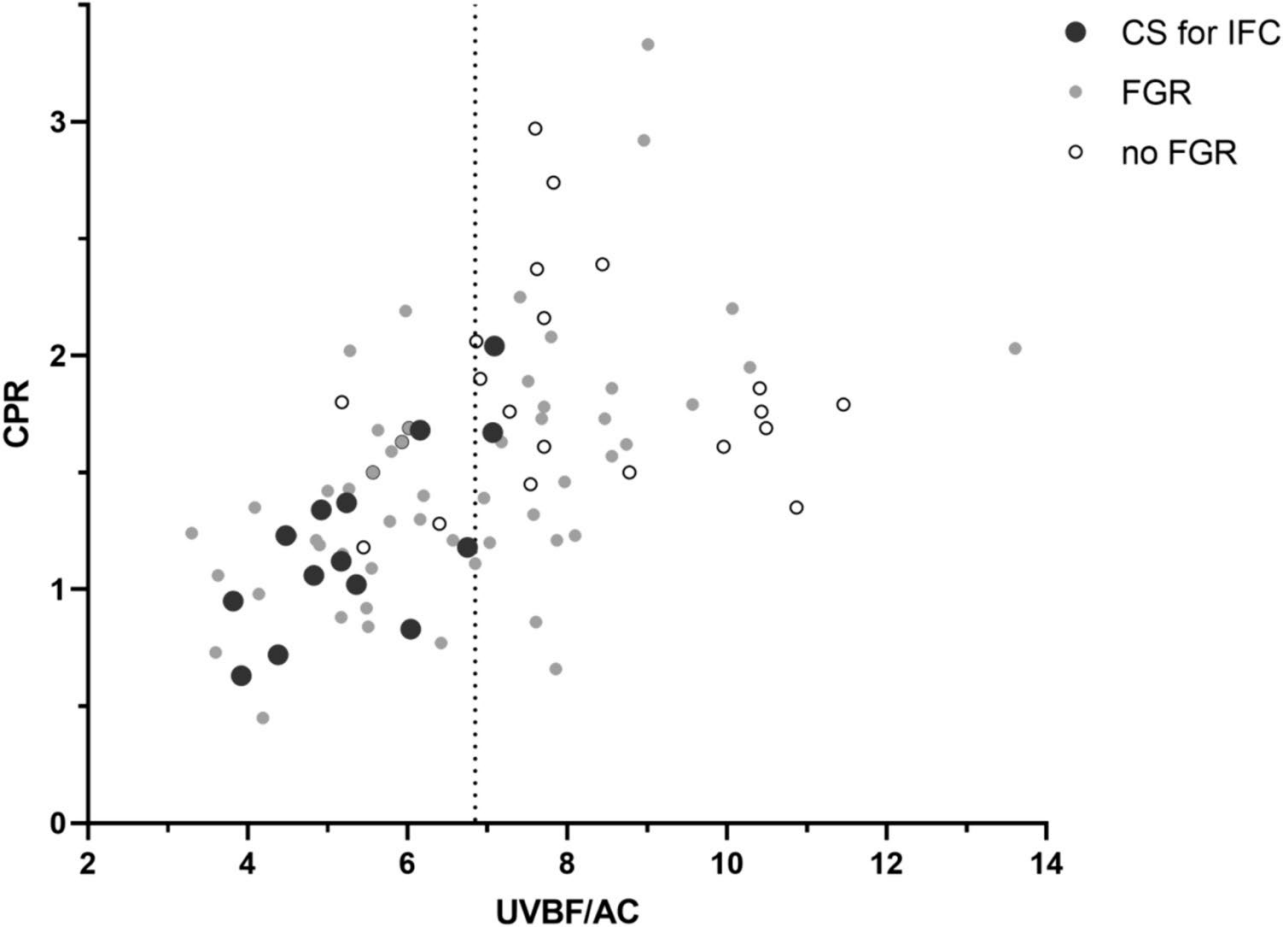
Individual data of patients with and without cesarean section for intrapartum fetal compromise. Each dot represents an individual patient in relation to the prenatal CPR and UVBF/AC. Black dots indicate those in whom cesarean section was performed for intrapartum fetal compromise (IFC). Filled black and gray dots represent fetuses that met the Delphi criteria [1] for definition of fetal growth restriction (FGR). Empty dots (o) represent fetuses that did not fulfill the Delphi criteria and were labeled as no FGR. Dotted line indicates the cut-off value for UVBF/AC at 6.85 (50th centile). *UVBF* umbilical vein blood flow, *AC* abdominal circumference, *CPR* cerebroplacental ratio

A cut-off value set at the 25th centile of UVBF/AC reached a sensitivity of 57% and specificity of 81% (Table 5). Patients with a prelabor UVBF/AC of less than the 50th centile was associated with an 8.1-folded risk for CS due to IFC (95% CI, 1.7–37.8), with a sensitivity of 86% and specificity of 58%. Individual data are shown in Fig. 2. Based on the Youden index [21], 7.135 was calculated as the optimal cut-off value. None of the 37 deliveries with UVBF/AC values ≥ 7.135 was complicated by cesarean section for IFC, resulting in 100% sensitivity and 51% specificity. This cut-off might be helpful to reassure a woman who would like to deliver vaginally. When applying the current definition of FGR based on Delphi consensus on our study cohort [1], all women with fetuses at risk of cesarean section for IFC could be identified at the cost of a poor specificity of 32%. CPR ≤ 1 was not significantly associated with cesarean section for IFC.

**Table 5:** Test characteristics for predicting cesarean section for intrapartum fetal compromise

Variable	Cut-off	n	CS for IFC	Sensitivity (%)	Specificity (%)	PPV (%)	NPV (%)	OR [95% CI]
								p value
CPR	≤ 1	13	4	29	88	31	86	2.8 [0.8; 10.8]
								p = 0.118
FGR	Delphi criteria†	64	14	100	32	22	100	NA
UVBF/AC	< 5.27 (25th centile)	21	8	57	82	38	91	6.2 [2.0; 19.4]
								p = 0.002
	< 6.85 (50th centile)	43	12	86	58	28	95	8.1 [1.7; 37.8]
								p = 0.003
	< 7.135^*^	50	14	100	51	28	100	NA

Data are expressed as odds ratios (OR) and 95% confidence interval [CI]. p values calculated using χ^2^ test

^†^Delphi criteria for late FGR ≥ 32 weeks: AC/EFW < 3rd centile or both AC/EFW < 10th centile *and* CPR < 5th centile or UA PI > 95th centile

^*^derived by Youden-index[21]

*AC* abdominal circumference, *CPR* cerebroplacental ratio, *CS* cesarean section, *EFW* estimated fetal weight, *FGR* fetal growth restriction, *IFC* intrapartum fetal compromise, *NA* not applicable, *PPV/NPV* positive/negative predictive value, *UVBF* umbilical vein blood flow

We further characterized the relationship of UVBF to other parameters. Correlation analyses revealed positive correlations between UVBF and EFW (r = 0.41, p < 0.0001) and AC (r = 0.33, p = 0.0017). UVBF was also positively correlated to CPR (r = 0.62, p < 0.0001) and MCA PI (r = 0.50, p < 0.0001). A negative correlation was found between UVBF and UA PI (r = -0.38, p = 0.0003) and AoI PI (r = -0.23, p = 0.03) (Table 6). As UVBF was not correlated with gestational age, we have reported UVBF as absolute values and corrected for EFW and AC. Additionally, neither mod-MPI (r = 0.07; 95% CI -0.15–0.28, p = 0.53) nor AoI PI (r = 0.07, 95% CI -0.15–0.28, p = 0.54) were correlated with gestational age.

**Table 6:** Correlation of absolute umbilical vein blood flow to Doppler and fetal parameters

Variable	Spearman correlation coefficient r [95% CI]		p value
EFW	0.41	[0.21; 0.58]	< 0.0001
AC	0.33	[0.12; 0.51]	0.002
CPR	0.62	[0.46; 0.74]	< 0.0001
MCA PI	0.50	[0.32; 0.65]	< 0.0001
UA PI	−0.38	[−0.55; −0.17]	0.0003
AoI PI	−0.23	[−0.43; −0.02]	0.03
mod-MPI	0.06	[−0.16; 0.27]	0.57
Gestational age at examination	0.03	[−0.19; 0.25]	0.76
pH umbilical artery	0.06	[−0.16; 0.27]	0.57
APGAR 5 min	−0.11	[−0.32; 0.11]	0.29

*AC* abdominal circumference, *AoI* aortic isthmus, *CPR* cerebroplacental ratio, *CS* cesarean section, *EFW* estimated fetal weight, *MCA* middle cerebral artery, *mod-MPI* modified myocardial performance index, *PI* pulsatility index, *UA* umbilical artery, *UVBF* umbilical vein blood flow

## Discussion

We explored the association between Doppler ultrasound parameters of fetuses with an EFW < 10th centile near term and the risk of cesarean delivery for IFC. We found (i) that fetuses delivered by cesarean section for IFC exhibited significantly lower levels of UVBF (both absolute and corrected for EFW and AC) and lower levels of VAI prior to onset of labor; (ii) that UVBF/AC was moderately accurate in predicting cesarean delivery due to IFC; and (iii) that none of the Doppler parameters were associated with neonatal acidosis.

Identifying those fetuses with impaired growth among the SGA fetuses is challenging prenatally, but would be crucial in order to improve perinatal outcome. Particularly, in late-onset growth-restricted fetuses, UA PI remains normal despite the development of brain sparing [3]. Conversely, multiple studies have demonstrated that reduced CPR and MCA PI levels are linked to fetal compromise during labor, low neonatal arterial blood pH, and admission to the neonatal unit [7, 22].

The present study confirms the existing literature on UVBF/AC as a promising marker to aid clinical risk stratification and patient counseling on the place and mode of delivery [4, 7, 23, 24]. In our cohort, the calculated area under the curve of UVBF/AC (AUC 0.78) was comparable to previously reported AUCs of up to 0.72 [7, 23]. In contrast to the umbilical vein blood flow, CPR was not significantly associated with cesarean delivery for intrapartum fetal compromise. This may not be surprising, as UVBF represents a more physiological measurement of placental function and the quantity of nutrients and oxygen reaching the fetus [4, 13, 16]. We corrected UVBF for the abdominal circumference to emphasize the importance of a low AC on perinatal outcomes. As a result, our analyses support the direct impact of insufficient umbilical vein blood supply on fetal susceptibility to intrapartum stress with subsequent need for operative delivery in SGA fetuses near term [25]. Reacting to intrapartum hypoxia is primarily depending on the clinical situation and how to end labor most effectively to avoid further hypoxic damage. It is widely known that with advancing labor, especially the second stage is associated with the highest risk of hypoxia. In fact, labor models in sheep showed a decrease in fetal scalp pH during the first stage of labor of 0.016 pH per hour and 0.11–0.12 pH units per hour during second stage [26]. For those high-risk fetuses that cannot even endure the comparably moderate stress during the first stage of labor, delivery by cesarean delivery is often the only possibility. As reported by Prior et al. our analyses showed the lowest umbilical vein blood flow in infants born by cesarean for intrapartum compromise [24]. We demonstrated a significant difference in UVBF/AC between the fetuses delivered by cesarean due to IFC compared to the group with a spontaneous vaginal delivery, while direct comparisons to infants with an instrumental delivery for IFC did not reveal any significant variation [24]. We assume that an impaired umbilical vein blood flow identifies those highly susceptible fetuses with a high risk of an early exhaustion of their bases reserves, achieving progressively a lower tolerance to hypoxic insults, which necessitates cesarean delivery. If a patient choses to delivery vaginally, identification of fetuses with an impaired prelabor umbilical vein blood flow may necessitate more intensive intrapartum monitoring and early recourse to operative delivery should any concerns arise.

However, implementing UVBF into clinical practice has been hampered as inter- and intraobserver reproducibility and feasibility of the measurement have been criticized [7]. To overcome errors, we reported both absolute values and UVBF corrected for EFW and AC. Furthermore, all measurements were conducted by one single experienced examiner to ensure consistency. In addition, median blood flow rate in the UV in our group of cesarean deliveries for IFC was 153.6 ml/min and comparable to the values of 151.2 ml/min reported by Parra-Saavedra et al. in their group of non-reassuring fetal state in term SGA fetuses [4].

Although the fetal aorta and the myocardial performance index represent the balance between the myocardium and the systemic and cerebral blood circulation, we failed to detect an association of these markers with operative delivery due to IFC. While some studies found strong correlations of abnormal Doppler imaging of the fetal aorta with adverse perinatal outcome [8, 27], others failed to show an additional clinical benefit for the perinatal management of SGA and FGR fetuses [28]. Studies on fetal cardiac function detected subclinical cardiac dysfunction among SGA fetuses [11] with reduced cardiac output as an early sign of placental insufficiency [12]. Progressive fetal compromise seems to be accompanied by increasing mod-MPI values and elevated markers of cell damage such as brain natriuretic peptide [10]. The current literature is inconclusive on the additive predictive value of mod-MPI for adverse perinatal outcome in SGA fetuses [9, 29].

None of the Doppler parameters we examined were associated with neonatal acidosis. Although deterioration of umbilical artery pH indicates fetal compromise during labor, obstetric measures in response to abnormal heart rate patterns such as cessation of oxytocin until delivery influence the incidence of adverse neonatal outcomes. Severe neonatal acidosis < 7.1 only developed in five neonates, with only one case of base excess below 12 mmol/l, limiting the validity of Doppler parameters for these endpoints in our study.

A major strength of our study is most certainly the intensive ultrasound examination of multiple arterial and venous Doppler parameters in one study cohort to visualize fetal circulatory status in both systems simultaneously. Most other trials assess one particular fetal vessel or Doppler parameter and their association with perinatal adverse events. We are aware of the limited size of our study cohort, rendering the results rather explorative and more hypothesis-generating than confirmative. However, our prospective, blinded design and the mostly homogeneous study population according to baseline characteristics and gestational age at examination constitute further strengths of this study. Time intervals between ultrasound examination and delivery were overall short and can therefore serve as adequate representation of fetal well-being before onset of labor.

Reduced umbilical venous blood flow is associated with an increased risk of secondary cesarean section for IFC in SGA fetuses near term. Our findings support the significance of assessing UVBF and will hopefully stimulate further research concerning implementation of UVBF into clinical practice to accurately identify those patients at high risk for IFC and to allow for better individualized patient counseling.

### Supplementary Information

Below is the link to the electronic supplementary material.

Supplementary file1 (PDF 15 KB)

## Data Availability

The authors can share the data upon a reasonable request by editors. However, the data cannot be shared routinely according to the legal regulations in our country.
