# Supplementary material for: Association between Doppler assessment and secondary cesarean delivery for intrapartum fetal compromise in small-for-gestational-age fetuses
Source: Arch Gynecol Obstet. 2024 May 24;310(2):719–28. doi: 10.1007/s00404-024-07559-2 (PMC11258169; doi:10.1007/s00404-024-07559-2)
Supplement: Supplementary file 1 — Supplementary file1 (PDF 15 KB) [file 404_2024_7559_MOESM1_ESM.pdf]

Doppler assessment for predicting secondary cesarean delivery for intrapartum fetal compromise in small-for-gestational age fetuses

Supplemental Table 1 Fetal Doppler parameters according to operative delivery and intrapartum fetal compromise.

	Instrumental vaginal delivery for IFC			Any operative delivery for IFC		
**Outcome**	**Yes (n=8)**	**No (n=79)**	**p value**	**Yes (n=22)**	**No (n=65)**	**p value**
UVBF absolute	205.9 (162.8-306.1)	204.5 (156.3-239)	0.531	165.7 (143.7-199.8)	220.3 (169.3-241)	0.01
UVBF/EFW	82.6 (73.3-116.2)	81.5 (64.7-98.2)	0.569	73.1 (59.5-90.4)	86.5 (67.2-103.4)	0.054
UVBF/AC	6.99 (5.7-9.8)	6.9 (5.2-7.9)	0.557	5.8 (4.7-7.1)	7.2 (5.5-8.3)	0.018
VAI	103.5 (75.1-160.5)	85.8 (61.3-113.2)	0.178	76.8 (52.5-100.4)	93.5 (68.0-115.4)	0.096
AoI PI	1.76 (1.59-1.97)	1.78 (1.57-1.01)	0.611	1.83 (1.66-2)	1.78 (1.56-2.02)	0.682
UA PI	0.8 (0.66-1.0)	0.92 (0.79-1.06)	0.085	0.95 (0.77-1.16)	0.92 (0.79-1.06)	0.589
MCA PI	1.31 (1.09-1.65)	1.32 (1.07-1.61)	0.997	1.18 (1.05-1.54)	1.39 (1.15; 1.62)	0.143
CPR	1.7 (1.3-2.1)	1.4 (1.2-1.8)	0.232	1.3 (1.1; 1.7)	1.5 (1.2-1.9)	0.15
mod-MPI	0.62 (0.56-0.73)	0.61 (0.56-0.71)	0.854	0.64 (0.60-0.76)	0.6 (0.55- 0.71)	0.098

Data are presented as median (interquartile range). P values were calculated using Fisher exact test for each Yes versus No. To adjust for multiple comparisons, Bonferroni correction was applied for each column. Alpha values <0.0056 ($P < 0.05/9$) were considered statistically significant (boldface).

AC: abdominal circumference; AoI: aortic isthmus; CPR: cerebroplacental ratio; CS: cesarean section; EFW: estimated fetal weight; IFC: intrapartum fetal compromise; MCA: middle cerebral artery; mod-MPI: modified myocardial performance index; PI: pulsatility index; UA: umbilical artery; UVBF: umbilical vein blood flow; VAI: venous-arterial index (UVBF [ml/min/kg]/UA PI).

Corresponding author: anna.scholz@med.uni-heidelberg.de
